# Supplementary material for: Burden and risk factors of snakebite in Mopeia, Mozambique: Leveraging larger malaria trials to generate data of this neglected tropical disease
Source: PLoS Negl Trop Dis. 2023 Aug 17;17(8):e0011551. doi: 10.1371/journal.pntd.0011551 (PMC10464960; doi:10.1371/journal.pntd.0011551)
Supplement: Attachment — Submitted filename: OBryan et al. Answers to reviewers.docx [file pntd.0011551.s001.docx]

Thank you for the opportunity to submit a revised manuscript.

**Reviewer #1**

| **Comment** | **Answer** | **Actions** |
| --- | --- | --- |
| Under “envenomation definition” – “Envenomation is when venom is injected into the victim during the bite, when venom is not injected it is a dry bite. To differentiate between dry bite and envenomation we assumed that more severe outcomes were due to envenomation.”  The above statements are confusing and not scientifically sound. Envenomation is defined as the development of clinical effects as a results of injecting snake venom to a person. Dry bite is defined as lack of clinical features developed when there is a bite following a snake evident by fang marks. Dry bites and envenomation are not differentiated by development of more severe outcomes. This section has to be rewritten according to the accepted definitions and if this has affected the data analysis, data analysis also has to be corrected. | We agree our definitions were a bit clumsy for this key paragraph. The main limitation is the relative shortness of the questionnaire as no questions about fang marks or duration of symptoms were included.  We have expanded this section to explain better how missing school/work or incomplete recovery were used as proxies for systemic signs/symptoms, hence envenomation.  This is also addressed explicitly in the limitations section | Lines 172-178 have been corrected as follows:  *Snakebite is a bite by any snake. Envenomation is the development of local or systemic signs and symptoms. Snakebite without envenoming or “dry bite” is the absence of signs or symptoms in presence of fang marks. [17] Given the short length of the questionnaire, for the purpose of analysis, we used missing time from school/work or incomplete recovery at the time of the questionnaire as proxies for systemic signs/symptoms and participants with these findings were accounted for as envenomation while those not reporting missing school/work or any sequelae at the time of questioning were accounted for as dry bites.* |
| I wonder whether the investigators have collected other known risk factors for snakebite such as type of occupation, specific agricultural activities and some details of snakebite such as type of snake bitten by, site of bite (anatomical location), type of activity while snakebite and time of day when the snakebite happened. These factors are considered as well-known risk factors for snakebite in snakebite prevailing areas. | We confirm that the questionnaire did not include occupation (albeit 80% of the population of Mopeia are subsistence farmers) or type of snake.  We have however, following a comment from reviewer #2 added a list with the most common snake species present in Zambezia.  Equally, we did not collect the time of the day when bites happened, but we did collect the location (at home, on the yard, on the road), This is described in results under “place of biting” | Further demographic details were added in the methods section **lines 110 to 120** and the new table 1.  We have added now **table 2** with the snake species that are most likely prevalent in Mopeia. |

| **Comment** | **Answer** | **Actions** |
| --- | --- | --- |
| Since this paper has presented some outcome data, I wonder whether the study has collected treatment seeking data, type of treatment they received, practice of first aid. | We confirm we did not collect data on first aid or the type of treatment received. | We have further expanded the limitation section to reflect this. Lines 429-431  *As we used proxies for severity to equate envenomation our estimations may not be fully accurate. Furthermore, no data on suspected species of snake, first aid practices or the of treatment received was collected.* |
| It is highly recommended to include these well-known risk factors and other data in this type of risk factor analysis paper. | We agree, however, the nested nature of this study poses limitations to the extension and detail of the data collection. | We have further expanded the limitation section to reflect this. Lines 453-457:  *This severely limited the length of the questionnaire that could be dedicated to snakebite, we acknowledge this resulted in several open questions, nonetheless, given the scarcity of empirical data on snakebite burden in rural Mozambique, leveraging larger studies addressing better funded topics such as malaria has yielded valuable data that otherwise would not be available today.* |
| **Methods & results** |  |  |
| Page 12, Sociodemographic data: “The median age of the population was 15.6 (IQR 7.3 – 28.0). This indicate most of the subjects were in younger age. Please include the range of the age as well. | This refers to the age of the 70,831 individuals included in the demographic survey.  Yes, Mopeia has a young age structure given its high natality rate. The ages range from newborn to 100. Summary statistics are appropriate here to describe this large population. | We have expanded this section with a new reference for those with interest in further demographic data from Mopeia.  *See Ruiz Castillo et al. Malaria Journal 2023* |

| **Comment** | **Answer** | **Actions** |
| --- | --- | --- |
| “50·6% of the population were 15 years old or under, 43·7% were of working age (16-55 years). 50·9% were female, 57%....” Above two sentences have started with a numeric value, please re-word these two sentences to start with words and not by numeric value. Starting sentences by a numeric value has been observed in many sentences throughout the manuscript, suggest to re-word those sentences to begin with words. | Thank you. This paragraph has been rephrased and the full text reviewed to avoid phrases starting with numbers. | Revised throughout |
| Pahe 12, General snakebite data “Between 16·9 and 74·8% of bites resulted in envenoming, depending on the criteria used for defining envenomation (incomplete recovery or days of work/school lost). The resulting incidence is 70·9 to 313·6 envenomation’s per 100,000 persons per year” If it is reporting incidence and envenomation percentage of the study population, how it can appear as a range (16.9 – 74.8% envenomation and 70.9 – 313.6 incidence)? Percentage of envenomation should be present as the value of number of patients developed clinical effects divided by total number of snakebites. | Thank you. We agreed the way this was presented was prone to causing confusion. We have now combined both criteria and show a unified result. | See lines 213-219:  *Using missed school/work days and incomplete recovery at the time of the study, 210 (77%) of the bites resulted in envenomation. The resulting incidence is 296 envenomations per 100,000 persons per year. All those who were bitten survived, 17.3% reported not making a full recovery at the time of survey.* |
| The word “envenoming” has been used in some places and “envenomation” is used in other places. Please use uniform abbreviation to describe this throughout the manuscript. | Thank you. We now use envenomation throughout | Revised throughout |

| **Comment** | **Answer** | **Actions** |
| --- | --- | --- |
| “All those who were bitten survived, 16·9% reported not making a full recovery at the time of survey.” – How did you access the full recovery? This means some patient may has clinical effects even 12 months after the bites. These persistence of clinical effects data has to be presented; what % of patients had not fully recovered after 1 month, 3 months, 6 months, 9 months and 1 years after the bite. | The questionnaire was conducted retrospectively at population level. One of the potential answers to the question about outcome of the bite was “partial recovery”, operationally, participants were asked if they were still suffering any consequences from the bite. With this array, it is not possible to answer your comment about duration of recovery. | The snakebite questions are now included as ***table 3*.** |
| Page 12, Demographics of those bitten “Just under half of those bitten were female…” Earlier it indicates that 50.9% were females. If 50.9% is the correct figure, it should not be “just under half…” | The 50.9% refers to the general proportion of females in the population of 70,831 surveyed.  The proportion of snake bites in women was 47.8% (130/272), making it just under half | Line 198-9 has been revised as follows:  *The median age of the population* ***included in the survey*** *was 15.6 years (IQR 7.3 -28.0).* |
| In table 1: In what basis did you defined these age categories; <16, 16-55 and >55? Weather the values are significantly difference or not depends on how the age categories were defined. These age categories should be made equally such as >10, 10-19, 20-29, 30-39 and so on. | We have changed the groups to <15, 15-64 and >64. 15-64 is generally working age and given snakebite can be considered an occupational hazard in rural areas like Mopeia we thought this would be the most helpful grouping as this population might be particularly at risk.  Please note the odd ratios and the related p-values are calculated in the rate per 1000 individuals, not the raw numbers, hence, even with unequal sizes, these differences remain. | We have added a column with the rate per 1,000 individuals to ***table 4.***  We also present a bar chart with bite incidence by age group in increments of 5. ***Figure 2*** |
| Figure 2: Suggest to include the months average rainfall in mm in right y axis of the graph. | Thank you for this suggestion. | We have included the precipitation data in the legend of this graph, now ***figure 3***. |
| **Conclusion** |  |  |
| Conclusions are drawn in to certain state based on the gathered data. I feel that collected data set is not comprehensive to draw the conclusions according to the title and objective of the study. | We have expanded the definitions and limitation section. We believe these changes make clear on what grounds conclusions are made. We aimed at providing an estimate on snakebite burden in one district in rural Mozambique, we surveyed over half the district to provide this estimate. No over-stated conclusions are presented. |  |

| **Comment** | **Answer** | **Actions** |
| --- | --- | --- |
| **Editorial and data presentation modifications** |  |  |
| (none) | -- | -- |
| **Summary and General Comments** |  |  |
| Objective of this study has not been properly mentioned before the Method section. | Thank you, this has been added | See lines 96-98 *The primary objective of this work was to provide local decision makers with descriptive data on the burden of snakebite in Mopeia, a rural district in Mozambique; this includes the incidence of snakebite envenomation collected retrospectively through a community survey.* |
| Language has to be improved before acceptance | The full manuscript has been reviewed by a native English speaker | Revised throughout |
| Some of the most important demographic, socioeconomic, risk factors have not been collected and presented in the paper. | Yes, this has been addressed in answering your previous comments on the general and methods sections. | See ***methods*** and ***limitations*** |

**Reviewer 2**

| **Comment** | **Answer** | **Actions** |
| --- | --- | --- |
| The objectives of this nested study is clearly stated. The study design is not the ideal as this is a spin-off of a malaria study. However, the data presented is important. the population is clearly described. the authors have used appropriate statistical tests to present the data. there are no concerns regarding ethics of the study | Thank you for your review |  |
| **Methods & results** |  |  |
| Analysis matches the plan. Results clearly presented. Figures and tables are of acceptable quality | Thank you |  |
| **Conclusion** |  |  |
| The conclusions are based on the results. Authors have clearly identified the limitations of the study and public health relevance has been addressed | Thank you |  |
| **Editorial and data presentation modifications** |  |  |
| It is advisable to include a list of snakes in the area along with their respective reported mortality and morbidity rates. | Thank you for this valuable comment. We have now included a table with the most frequent snakes described in the area | See ***table 2***. |
| It would also be useful to look in to hospital records to get an idea about the mortality of snake envenoming in this region. | We agree with this comment and believe this work provides a good base to built upon on snakebite research in the region. | We have added this to lines 308:  *Additionally, it would be valuable to examine the reasons for the lack of mortality in Mopeia as well as to conduct a review of the local hospital records* |
| **Summary and General Comments** |  |  |
| This is an " opportunistic" study that has resulted in generation of some useful data. Even though, the methodology is not the best to describe snakebite epidemiology, the authors have been honest about its limitations. | Thank you |  |

**Reviewer 3**

| **Comment** | **Answer** | **Actions** |
| --- | --- | --- |
| Thanks for the opportunity to review this very interesting and useful article. It is great to have more data on SBE in Mozambique. However, I have found a few important issues to discuss, probably deserving a relatively major review, despite being a very important paper:  Before going into the Methods/Results I would like to emphasize that: | Thank you for your review |  |

| **Comment** | **Answer** | **Actions** |
| --- | --- | --- |
| The introduction chapter and the short Reference list show a certain lack of analysis (or experience) about the impact of snakebite envenoming in Mozambique. You have not mentioned the multiple reports on NTDs including SBE, by the Mozambican Ministry of Health (NTD dpt). The MoH reports clearly discuss the problem of snakebite ("cobras") in Mozambique, including facility-based data,which were shared with WHO's NTD Dpt, and contribute to the WHO global NTD Roadmap, and WHO Snakebite Roadmap to halve SBE deaths & disabilities by 2030. | Thank you.  The introduction has been revised and several new references have been included. | See new references:  5. Halilu S, Iliyasu G, Hamza M, Chippaux JP, Kuznik A, Habib AG. Snakebite burden in Sub-Saharan Africa: estimates from 41 countries. Toxicon. 2019 Mar 1;159:1–4.  6. Farooq H, Bero C, Guilengue Y, Elias C, Massingue Y, Mucopote I, et al. Snakebite incidence in rural sub-Saharan Africa might be severely underestimated. Toxicon. 2022 Nov 1;219:106932.  8. Chaccour C, Casellas A, Hammann F, Ruiz-Castillo P, Nicolas P, Montaña J, et al. BOHEMIA: Broad One Health Endectocide-based Malaria Intervention in Africa—a phase III cluster-randomized, open-label, clinical trial to study the safety and efficacy of ivermectin mass drug administration to reduce malaria transmission in two African settings. Trials [Internet]. 2023 [cited 2023 Jun 15];24(1). Available from: /pmc/articles/PMC9942013/  9. Instituto Nacional de Estatistica - Moçambique. QUADRO 52. POPULACAO POR TIPO DE HABITACAO E SEXO- SEGUNDO PROVINCIA E AREA DE RESIDENCIA. PROVINCIA DE ZAMBEZIA- 2017 - INE [Internet]. 2023 [cited 2023 Jun 20]. Available from: <https://www.ine.gov.mz/web/guest/d/quadro-52-populacao-por-tipo-de-habitacao-e-sexo-segundo-provincia-e-area-de-residencia-provincia-de-zambezia-2017>. Portuguese.  10. Ruiz-Castillo P, Imputiua S, Xie K, Elobolobo E, Nicolas P, Montaña J, et al. BOHEMIA a cluster randomized trial to assess the impact of an endectocide-based one health approach to malaria in Mozambique: baseline demographics and key malaria indicators. Malaria Journal [Internet]. 2023 Jun 4 [cited 2023 Jun 15];22(1):1 Available from: <https://malariajournal.biomedcentral.com/articles/10.1186/s12936-023-04605-3>  11.  12. 12. Chaccour C, Zulliger R, Wagman J, Casellas A, Nacima A, Elobolobo E, et al. Incremental impact on malaria incidence following indoor residual spraying in a highly endemic area with high standard ITN access in Mozambique: results from a cluster‐randomized study. Malar J [Internet]. 2021 Dec 1 [cited 2023 Jun 15];20(1):1–15. Available from: https://malariajournal.biomedcentral.com/articles/10.1186/s12936-021-03611-7  13. 13.Longbottom J, Shearer FM, Devine M, Alcoba G, Chappuis F, Weiss DJ, et al. Vulnerability to snakebite envenoming: a global mapping of hotspots. The Lancet [Internet]. 2018 Aug 25 [cited 2022 Apr 26];392(10148):673–84. Available from: <http://www.thelancet.com/article/S0140673618312248/fulltext>  16. World Health Organization (WHO). Guidelines for the production, control and regulation of snake antivenom immunoglobulins, Annex 5, TRS No 1004. WHO Technical Report Series. 2013;(964):192.  17. Pucca MB, Knudsen C, Oliveira IS, Rimbault C, Cerni FA, Wen FH, et al. Current Knowledge on Snake Dry Bites. Toxins (Basel) [Internet]. 2020 Nov 1 [cited 2023 Jun 15];12(11). Available from: /pmc/articles/PMC7690386/  21 Chippaux JP. Estimate of the burden of snakebites in sub-Saharan Africa: A meta-analytic approach. Toxicon [Internet]. 2011 Mar 15 [cited 2021 Nov 10];57(4):586–99. Available from: <https://www.sciencedirect.com/science/article/abs/pii/S0041010111000055?via%3Dihub>  22. Mackessy SP. Handbook of venoms and toxins of reptiles. 2010.  23.  23. WHO Regional Office for Africa. Guidelines for the prevention and clinical management of snakebite in Africa. World Health Organization. Regional Office for Africa. WHO regional office for Africa [Internet]. 2010;1–145. Available from: <https://apps.who.int/iris/handle/10665/204458>  27. Musah Y, Ameade EPK, Attuquayefio DK, Holbech LH. Epidemiology, ecology and human perceptions of snakebites in a savanna community of northern Ghana. PLoS Negl Trop Dis [Internet]. 2019 [cited 2022 Apr 27];13(8):e0007221. Available from: https://journals.plos.org/plosntds/article?id=10.1371/journal.pntd.0007221 |
| You do not mention the very important Mozambican community-based epidemiological study by Farooq et al (Toxicon 2022) in Cabo Delgado which found more snakebites (n=297) and many more deaths (n=46). | Thank you. This report was written before the publication of Farooq et al. This is nonetheless no excuse for not being up to date on the topic. We have read and cited this important piece. | We have now cited this study 7 times (lines 86, 300, 317, 328, 346, 393, 459) (reference number 6) |
| You did not adjust your objectives taking into account their opinions (did you talk to them?) - you should mention previous epi work on SBE by Mozambican and other African experts. | We confirm Dr. Marilia Massangaie and Dr. Henis Sitoe, the past and current managers of the NTD program of Mozambique are aware of the NTD component of the BOHEMIA trial.  We have now read and cited several additional SBE publications by African experts. | African snakebite experts  See references: 5. Halilu S, Iliyasu G, Hamza M, Chippaux JP, Kuznik A, Habib AG. Snakebite burden in Sub-Saharan Africa: estimates from 41 countries. Toxicon. 2019 Mar 1;159:1–4.  6. Farooq H, Bero C, Guilengue Y, Elias C, Massingue Y, Mucopote I, et al. Snakebite incidence in rural sub-Saharan Africa might be severely underestimated. Toxicon. 2022 Nov 1;219:106932.  18. Alcoba G, Chabloz M, Eyong J, Wanda F, Ochoa C, Comte E, et al. Snakebite epidemiology and health-seeking behavior in Akonolinga health district, Cameroon: Cross-sectional study. PLoS Negl Trop Dis [Internet]. 2020 Jun 1 [cited 2022 Mar 30];14(6):e0008334. Available from: <https://journals.plos.org/plosntds/article?id=10.1371/journal.pntd.0008334>  27. Musah Y, Ameade EPK, Attuquayefio DK, Holbech LH. Epidemiology, ecology and human perceptions of snakebites in a savanna community of northern Ghana. PLoS Negl Trop Dis [Internet]. 2019 [cited 2022 Apr 27];13(8):e0007221. Available from: <https://journals.plos.org/plosntds/article?id=10.1371/journal.pntd.0007221>  28. Ooms GI, van Oirschot J, Waldmann B, Okemo D, Mantel-Teeuwisse AK, van den Ham HA, et al. The Burden of Snakebite in Rural Communities in Kenya: A Household Survey. Am J Trop Med Hyg [Internet]. 2021 Sep 15 [cited 2021 Nov 12];105(3):828–36. Available from: <https://www.ajtmh.org/view/journals/tpmd/105/3/article-p828.xml>  References 8, 10 and 12 include African authors but not experts in snakebite. |
| Regarding partnership with Centro de Investigacao em Saude de Manhica ( en.cismmanhica.org ): was there any preliminary work on snakebite, e.g. discussions with MOH NTD dpt , estimation of sample size based on previous data, or with Farooq et al.? | The malaria trial used as a platform for collecting this data has been inn planning since 2017. The sample of the SBE component comes from the preceding demographic survey, which included approximately 50% of the whole Mopeia district. Our previous interactions include both NTD program managers as well as the NDT focal person for USAID. These interactions occurred as early as 2017 and precede Farooq et al by years. Beyond punctual reports, we were not able to obtain reliable SBE epidemiological data from rural Mozambique through 2017-2019. | |

| **Comment** | **Answer** | **Actions** |
| --- | --- | --- |
| 1) The main objective is clearly stated (cross-sectional prevalence = 12-month retrospective incidence/100 000 pop.). | Thank you. This has been included | This has been included at the end of the introduction in lines 96-98:  *The primary objective of this work was to provide local decision makers with descriptive data on the burden of snakebite in Mopeia, a rural district in Mozambique; this includes the incidence of snakebite envenomation collected retrospectively through a community survey.* |
| 2) The main methodological issue which is presented as an advantage (Nesting it within a malaria survey in 1 small district, with no previous sample estimation, or visible request by authorities) probably lead to 2 methodological issues: - It required a very small questionnaire (only 9 questions) not too overload the main malaria study - which was the priority probably. - The sampling method was not designed for a study focused on snakebite, multi-cluster, 2-stage sampling of clusters and households visibly done with a focus on children and distances adapted to malaria surveys. In contrast, by looking at the map, all clusters seem quite close the main road, not necessarily in the most remote (central) areas of the district (this is only a hypothesis, and possibly explaining the zero-death in the results). | We proposed a nested study that provided information on SBE in a rural district where no previous data on the subject existed. The opportunistic nature of the sampling allowed to leverage the resources destined to a large malaria study whose preparatory demographic phase included enumerating **100% of households**. The final sample size of households included in the full demographic survey consists in over half of the population of the whole district which provides enough power for an accurate estimation of the SBE burden.  Study clusters were randomly generated using the baseline of households enumerated (100% of them), there was no geographic stratification, nor where areas closer to roads favored, hence the sample is representative of Mopeia. There is no population in the center of the district which is an area with seasonal flooding.  We agree however that a short questionnaire with just 9 questions is sub-optimal, however, given the scarcity of empirical data on rural areas, studies such as ours do provide valuable information that otherwise would not be collected. | We have added further details on the geography and socioeconomic situation of Mopeia, including the fact that the south is scarcely populated given floodings to the methods section. Lines 111-113:  *The population is rural and disperse and the central/south floodplains have much lower population density than the northern part traversed by the national road N1*  We have added further details on the sampling strategy (random, not stratified): line 128-130:  *Creation of the clusters was not restricted or stratified by location or any other criteria, hence this sample is geographically representative of the district*  We have now included the full SBE questions asked in the methods as ***table 3***. |
| **Comment** | **Answer** | **Actions** |
| 3) The sample size could seem appropriate (70 000), but only 272 Snakebite victims and zero deaths were found. Sub analyses and multivariate (crude or adjusted) regressions could have suffered from the small samples in each subcategory. Again, how did you calculate your sample size - did you take in to account:  - the Mozambican MOH data on snakebite from the NTD department - you would be surprised by the numbers of "cobra" bites reported recently throughout the country. I read this report about 1-2 years ago but i can't find it now.  - the recent study by Farooq et al. (Toxicon 2022) on snakebite in Mozambique (!) - it found a very high incidence and included 1year, 5-yars, 10-years recall, in Cabo Delgado province. (sorry for the repetition, as I also mentioned this in the intro). | The sample size of the census includes 53% of the whole district.  We agree that the frequency of the outcome is low (272), this is appropriately mentioned in the limitations and conclusion.  However, this work is mostly descriptive, the main outcome is the incidence at population level and the power of that extrapolation is based on a large an appropriate sample consisting in over half a district surveyed.  Thanks for pointing to Farooq et al. We have indeed read and cited this now. | We have added further details on the sampling strategy in the methods section, as above.  The limitations of the models are mentioned under “generalizability” Lines 451-452  *The results of the sub-analyses and adjusted models should be interpreted carefully given they are based on 272 individual bites*  This is also highlighted in the conclusion.  Several additional references have been added as detailed above |
| 4) We are flattered that you were inspired by our 3 snakebite-epidemiology studies (Alcoba PLOS NTD, Alcoba Lancet GH, Babo Lancet GH) , in Cameroon and Nepal , including habitat & animal exposures (although animal snakebites and precise risk per animal type was not analysed), but you should have mentioned the very numerous studies conducted in Africa (recently I found about 50 references) plus epidemio data within WHO guidelines : https://www.who.int/publications-detail-redirect/9789290231684 (ISBN: 978 92 9 023168 4 WHO REFERENCE NUMBER: WHO/AFR/EDM/EDP/10.01). | We appreciate this reference and the many African-led works it contains. We have now read and appropriately cited several of these. | African snakebite experts  See references: 5. Halilu S, Iliyasu G, Hamza M, Chippaux JP, Kuznik A, Habib AG. Snakebite burden in Sub-Saharan Africa: estimates from 41 countries. Toxicon. 2019 Mar 1;159:1–4.  6. Farooq H, Bero C, Guilengue Y, Elias C, Massingue Y, Mucopote I, et al. Snakebite incidence in rural sub-Saharan Africa might be severely underestimated. Toxicon. 2022 Nov 1;219:106932.  18. Alcoba G, Chabloz M, Eyong J, Wanda F, Ochoa C, Comte E, et al. Snakebite epidemiology and health-seeking behavior in Akonolinga health district, Cameroon: Cross-sectional study. PLoS Negl Trop Dis [Internet]. 2020 Jun 1 [cited 2022 Mar 30];14(6):e0008334. Available from: <https://journals.plos.org/plosntds/article?id=10.1371/journal.pntd.0008334>  27. Musah Y, Ameade EPK, Attuquayefio DK, Holbech LH. Epidemiology, ecology and human perceptions of snakebites in a savanna community of northern Ghana. PLoS Negl Trop Dis [Internet]. 2019 [cited 2022 Apr 27];13(8):e0007221. Available from: <https://journals.plos.org/plosntds/article?id=10.1371/journal.pntd.0007221>  28. Ooms GI, van Oirschot J, Waldmann B, Okemo D, Mantel-Teeuwisse AK, van den Ham HA, et al. The Burden of Snakebite in Rural Communities in Kenya: A Household Survey. Am J Trop Med Hyg [Internet]. 2021 Sep 15 [cited 2021 Nov 12];105(3):828–36. Available from: <https://www.ajtmh.org/view/journals/tpmd/105/3/article-p828.xml>  In addition we have included further references from non-African snakebite experts and 2 additonal WHO snakebite reports. |

| **Comment** | **Answer** | **Actions** |
| --- | --- | --- |
| **Methods & results** |  |  |
| Yes analysis and results are rather clear. | Thank you |  |
| However we would have liked to have more complete data about severity/syndromes and access to health care (facility vs traditional care?) among many other potential multivariate analyses. | Yes, that would be desirable. This is however the second to date community survey on SBE conducted in Mozambique. For this, we have leveraged an existing malaria platform with the discussed limitations. | We have however now included ***table 5***, which provides details on the reported recovery by age group. |
| Open questions or focus groups could have been critical in *understanding* the main public health problems : footwear, occupational hazard, access to antivenom (and price), dangerous practices (in facility or traditional)... etc. | We agree. | Some of these suggestions, and the ones above have been added to the discussion as potential/desired future steps.  Lines 464-467  *further exploration on absence of reported deaths, a better understanding on the burden of snakebite on the local health system as well as possibly using qualitative methods to understand the perception of the public around this issue* |
| Clustering impact? effect is not presented. Do you have an estimate of adjusted incidence (cluster weighting?)- this is a specific analysis available in Stata or R. | The clusters were drawn for the malaria trial but not constrained by village limits. The survey was conducted in a population spanning beyond 50% of the district and not restricted by geography. |  |

| **Comment** | **Answer** | **Actions** |
| --- | --- | --- |
| FIG: Map shows bites on the roads  - any explanation? any clustering? space + time -> no obvious trend (all colours=months seem mixed without any specific pattern, did you find any. Any HOTSPOT in the district => FOCUS for next preventive measures + trainings + antivenom supply?  There is a light lack of spatial-epi analysis here. | Thank you for pointing this out. Yes, these dots reflect the households reporting bites. Population in Mopeia is concentrated along the primary and secondary roads.  We indeed created heatmaps that were not included in this submission given that they only reflect that more bites are reported in the area that concentrates 30% of the population, the district´s capital. We have now included these as supplementary material | We have expanded the results section under “Location and seasonality” as well as the discussion.  Lines 256-260  *Note that bites are frequently reported in households along the roads, this reflects the distribution of the population in Mopeia which is mostly clustered along primary and secondary roads. Heatmaps were constructed with these data (figure 5), these reflect a higher occurrence of snakebite in the most densely populated region, Mopeia Sede, which concentrates over a third of the district´s population.*   We have added the heatmaps (fig 5) |
| Some mistakes in the abstract:  risk of SB "increases with age" (false) : it decreases in the older population, in the >55yo group - please be precise on the most-affected category = as usual - risk = young age or working-age (adolescents? please separate from other children,  and young adults) .  An age-sex pyramid of victims vs participants could help summarise all of this precious info. - same mistake in Results chapter (incidence decreases with old age, peaks among the 16-50, this could be subdivided according to 4-5 cats, eg <5, 5-15, 15-25, 25-35, or 25-45, 45+) - check Table 1 - clarify which type of p-value?    Odds ratio decreases in 3rd category... | We divided the age groups to best reflect field work burden. We used 15-55 as the ages conducting most of field work.  Note 50% of the district is under the age of 16. In our analysis, there is an OR of 4.4 and 5.7 for ages 16-55 and > 55 when compared with under 16. So, the risk is indeed higher in ages 16 and above.  We have re-analized the data divided as 0-5, 5-<15, 15-64 and >64, the results remain unchanged: using the rate of bites per 1000 population-years in those 5-<15 as reference we see the following:  -The rate per 1000 in those 0-5 is 1/10 BUT, this is based in just 3 bites in under 5s.  -The rate in those 15-64 is 3-fold and,  -The rate in those >64 is 4.5-fold (the latter two are significant) | We have substantially modified ***table 4*** to include common demographic division of population age (<5, 5-15, 15-64 and >64). We provide the incidence crude and adjusted by population size of every age.  We also provide a bar chart with the number of bites per age group in increments of 5 (**Figure 2**) |
| **Comment** | **Answer** | **Actions** |
| Loss of limb : do you mean amputation? autoamputation due to necrosis ? or loss of "limb function" (disability without amputation). Total disability?(inability to walk?)... this is a bit vague. | Thank you. We have now added a table with all questions asked related to snakebite and included the operational definition used. | Please see ***table 3*** and its legend (lines 147-148):  *“Loss of limb” was operationally defined as amputation (medical or necrosis) or loss of function.* |
| FIG 2: Not easy to say if risk is higher during dry vs rainy season (almost same), especially with this small sample. I recommend a bit of caution, as usually rains+floods are well-known risk factors for snakebite in some places, but dry seasons can be linked to agricultural work with snakebite risk... this requires further analysis. | Yes. There are no strong conclusions on seasonality from our data with 52% occurring in the dry season, 42% in the rainy season and 5% unable to recall the exact month. The figure is just a bar chart to illustrate this, not to draw conclusions. | We have reworded the results section on seasonality (lines 391-394):  *No particular seasonality on the risk or severity of snakebite in Mopeia can be inferred from our data, as seen in Northern Mozambique and Nepal[6,28] and in contrast with Ghana and Kenya where bites are more common in the wet season.[20,27]* |
| Suggestion: when did the most severe bites occur (=those with more days of work/school lost , or those no/delayed recovery)? - during the dry/rainy - different per village? different according to distance to river/lake/forest/fields/ any known flood areas ... ? Further GIS analysis or simple paper map analysis possible? This is inter-linked with the above-mentioned clustering effect analysis and my previous question on clustering impact on DEFF (design effect). | We have analyzed the total and mean number of school/work days lost during the rainy season vs the dry season and found no differences.  We have also included a heat map as suggested. This map shows that snakebite is clustered around the district´s capital, where 1/3 of the population lives | The result section has been updated as follows (lines 273-274)  *There were no differences in the number of school/work days lost due to bites in the rainy season (total = 1,412, mean = 19.08 days) versus the dry season (total = 1,598, mean = 17.75) (t-tests, p = 0.8).*  See ***figure 5*** |

| **Comment** | **Answer** | **Actions** |
| --- | --- | --- |
| **Methods & results** |  |  |
| In your discussion-conclusions, I would like to see a little more in-depth appreciation of your study, compared to the Ministry's NTD dpt data on snakebite, and more comparisons with the other household survey on snakebite by Farooq et al. (Toxicon 2022), in a different part of Mozambique, but with very different findings: - Farooq's group found 297 SB victims and 46 deaths, within a smaller multicluster survey (7544 persons, 1037 HH). Incidence 352/100 000, similar to yours. BUT they "extrapolated that every year at least 69,261 people are victims of snakebite, of which at least 8950 result in death".(1/8) - How should you answer to this based on your (also quite local findings) , in terms of GENERALISABILITY? | As mentioned above, after consulting with the NTD department, publishing any national snakebite data requires ministerial approval. We have however included further references to Farooq et al as well as other relevant studies as suggested in other comments above. | We have included comments on the potential causes of this difference, see lines 459-467:  *However, this study occurred in a single district and despite wide geographical variation with main road in the north and flood plains in the south, including additional areas of Zambezia would have made the study more generalisable.[14] In practice, it would be impossible to conduct one study that accounted for all the variation seen in snake habitats and this is why each study on snakebite will be unique and an element of variation will always be present. Some of the questions left open following this study include: further exploration on absence of reported deaths, a better understanding on the burden of snakebite on the local health system as well as possibly using qualitative methods to understand the perception of the public around this issue.* |
| I would also like to read some of your hypothesis on the absence of mortality , in strong contrast with most other African studies (Mozambique and many other epi studies in Africa): -  Species variability (any idea which are the major snake species in Mopeia : Bitis, Naja, Dendroaspis, or  other less venomous... ? | Thank you. We have now included a table with the most common species expected in Mopeia. We also include several hypotheses around the low mortality reported in our study. | See Table 2 and lines 310-330  *Of the venomous snake species likely to be present in Mopeia (Table 2), the WHO regards Bitis arietans, Dendroaspis angusticeps, Dendroaspis polylepis and Naja mossambica to be most important in southern Africa.[23] Of these, D. polylepsis and D. angusticeps bite deliver potent neurotoxic venom often resulting in rapid death[14,23], as such we hypothesise that whilst their range includes Mopeia, it is unlikely many if any of our bites are as a result of these species. The higher morbidity seen in those bitten at home in this study is compatible with the presence of N. mossambica, an aggressive species known to enter houses and whose bites often result in severe injury but not usually rapid death.[6,14,23] The highly prevalent B. arietans accounts for a large proportion of snakebite morbidity across the world, causing severe injury but infrequent rapid death[14,23], and is almost certainly contributing to the burden in Mopeia (in fact, the BOHEMIA study team encountered – without harm! - a B.arietans during this data collection). In addition to these 4 important species, we hypothesise that in Mopeia and in neighbouring areas along the Zambezi River, Proatheris superciliaris, whilst being a category 2 species, could be responsible for a large proportion of the bites documented in our study. P. supercillaris has a very limited range (only found in pockets around Lake Malawi and Lake Chilwa and on the floodplains of the Shore and Zambezi Rivers)[13,14] meaning it appears infrequently in literature. There are no documented fatalities but it can cause severe symptoms,[14] in keeping with our high burden but zero fatalities. Similarly, Atractaspis bibronii found in Mopeia and across Africa, delivers severe but not fatal bites,[14] and was recently found to be the commonest cause of snakebite along with B. arietans in Cabo Delgado of northern Mozambique.[6] As such we consider N. mossambica, B. arietans, P. supercillaris and A. bibronii to likely be the snakes of most concern in Mopeia.* |

| **Comment** | **Answer** | **Actions** |
| --- | --- | --- |
| You were not looking in a particular hyperendemic area ("hotspot") ? | No. We have been conducting malaria research in Mopeia since 2016. Snakebite had never been flagged as a local public health problem. This is now mentioned in the manuscript | See lines 457-459:  *Additionally, this study was conducted in an area where snakebite had not been previously flagged as a public health problem, this is aligned with previous findings suggesting that the burden of snakebite in rural Mozambique is much higher than previously thought.[6]* |
| - You didn't go into the remote parts (far from roads) of the geographic area, remaining in less empoverished areas, (little influence of house constrution, income, ...) ? | This has been addressed in response to comments above. The sample size includes over half the district´s population and cluster creation was not stratified geographically. The sample includes all wealth strata from the richest and closest to the road to the remote most isolated poorer households. The absence of clusters in the middle of the district is aligned with very little population in the floodplains. | See also ***Figure 5*** |
| - Health seaking, good access to health (malaria treatments & bednets - correlation with snakebite risk)? | We have now included specific information about bed net usage. | See lines 246-249:  *Regarding the question “did you sleep under a bed net last night?”, 75% of the censed population responded positively. The proportion among those affected by snakebite was 82%, this difference was statistically significant. All but one of those bitten at home reported using a bed net on the previous night.* |
| - Could you compare the findings of your malaria study (not referenced nor explained) - and possible issues linked to double-burden. | We have provided an additional reference with information on the local burden of malaria and other diseases. The overarching malaria study has not been unblinded yet.  Additionally, further information on the malaria study and its results will not enrich the findings of this snakebite sub-study |  |

| **Comment** | **Answer** | **Actions** |
| --- | --- | --- |
| - One of the burning question for a few decades is the impact of bednets preventing snakebite at night, especially on children sleeping on the floor? Association of using bednets with less snakebites??? This would be a great added value of this double-malaria-snakebite nested study. --> Please try to inform the curious NTD reader on this possible association or absence of it? as you probably have good data on bednet, ACT/ivermectin access, etc - and links with socio-economic factors and occupational hazards. | We have added a paragraph with information on bed net usage in the general population and in those bitten by snakes. Please note that only 25 snake bites occurred at home. The apparent positive association between bed net usage the previous night and snakebite is highly likely to be confounded by other factors and the relatively small numbers of bites happening at home. | Results. See lines 246-249:  *Regarding the question “did you sleep under a bed net last night?”, 75% of the censed population responded positively. The proportion among those affected by snakebite was 82%, this difference was statistically significant. All but one of those bitten at home reported using a bed net on the previous night.*  Discussion. Lines 366-368:  *The bed net usage among those bitten at home was higher than in the general population but this finding is to be interpreted carefully given the small sample size.* |
| - Nesting : YES this is great as it allows a more affordable survey, which could certainly be repeated after your intervention (preventive, supply, etc). | Thank you. |  |
| - A bit of modesty, as your study is not the first in Mozambique, and MoH data had already highlighted that Mozambique was among the snakebite-endemic areas requiring investment in the NTD+SBE roadmaps. Bill Gates, Global fund & GAVI could include support. Few NGOs are already analysing and trying to implement integrated NTD projects, through other alliances, NNN, United to Combat NTDs, the Kigali Declaration, etc. | We acknowledge that failure to cite some work due to our inexperience in the snakebite field may be falsely interpreted as pride. We clarify that this report was written before the publication of Farooq et al.  This is nonetheless no excuse for not being up to date on the topic. We have read and cited this important piece together with several additional works from African Authors. |  |
| - I full agree that your study has an important value as "revealing" ... "gateway" towards an integration of Snakebite and NTDs among larger (better funded) projects, particularly malaria surveys. | Thank you for this comment. |  |

| **Comment** | **Answer** | **Actions** |
| --- | --- | --- |
| **Editorial and data presentation modifications** |  |  |
| - Very small (many snakebite epi studies and reports from MOH and WHO) missing | We have reached out to the MoH via the NTD program. The sharing of the report, particularly for citation and comparison of data in a publicly available manner requires ministerial clearance. | We have resorted to cite regional, rather than Mozambique specific data. |
| - Format of the reference seems incorrect: no DOI or PMID or standard PLOS-NTD reference style. | Thank you. | Reference style has been reviewed and corrected when required. |
| Minor edits: - Table 1: "effected" - do you mean affected? | Thank you. Indeed, this is a typo | This has been corrected in table 1 |
| Minor edits: - Loss of limb: please change to either "loss of limb function" (if correct) or "amputation" (if correct) otherwise it could be ambiguous. | This has been addressed in answer to one of the above comments. Please refer to table 3 for the operational definition of “loss of limb” used throughout the survey | Table 3 with the snakebite questions and the operational definitions of snakebite has been added  *“Loss of limb” was operationally defined as amputation (medical or necrosis) or loss of function.* |
| **Summary and General Comments** |  |  |
| Congratulations on this work,  but I really look forward to reading your further analysis, corrections, or clarification on a few major points. | Thank you for contributing to improve the quality of this work. |  |
